# Teacher Expectations and Parental Stress During Emergency Distance Learning and Their Relationship to Students’ Perception

**DOI:** 10.3389/fpsyg.2021.712447

**Published:** 2021-09-17

**Authors:** Ariana Garrote, Edith Niederbacher, Jan Hofmann, Ilona Rösti, Markus P. Neuenschwander

**Affiliations:** School of Education, University of Applied Sciences and Arts Northwestern Switzerland, Brugg, Switzerland

**Keywords:** distance learning, COVID-19 pandemic, school closures, parental stress, teacher expectations, student learning behavior

## Abstract

School closures in spring 2020 caused by the COVID-19 pandemic were an unprecedented and drastic event for students, parents, and teachers. The unplanned adaptation of classroom instruction to emergency distance learning was necessary to ensure continued education. In this new learning environment, teachers formed expectations for student academic achievement gains, which in turn affected the opportunities for students to learn. Parents faced new challenges in supporting their children’s learning. According to parenting stress models, such drastic events can be a stress factor for parents, which in turn affects their children’s adjustment. This study analyzed the extent to which parents and teachers affected the perceptions of students in compulsory school toward distance learning through processes at home (individual level) and at the class level with data from multiple informants. On an individual level, the relationship between parents’ perceived threat of COVID-19 and their stress due to distance learning and students’ perceived threat of COVID-19 and their perception of distance learning were examined. Students’ learning behavior was accounted for as a variable related to their perception of distance learning. At the class level, the explanatory character of teacher expectations and class-aggregated achievement gains were examined. Data on students in grades 4 to 8, parents, and teachers in Switzerland were collected with standardized online questionnaires after the period of school closures. A subsample of 539 students, 539 parents, and 83 teachers was analyzed. The results of multilevel structural equation modeling suggested that students had a more positive perception of distance learning if they were able to learn more autonomously (i.e., more motivated and concentrated than in regular classroom instruction) and if their parents felt less stressed in the distance learning setting. Parents were more stressed if they perceived COVID-19 as a threat. Students’ perception of the COVID-19 threat was related to their parents’ perception but did not explain students’ learning behavior. At the class level, if teachers expected high academic achievement gains in distance learning, the average academic achievement gains of a class were greater. The greater the achievement gains were, the more positive the collective student perception of distance learning was.

## Introduction

In response to the COVID-19 pandemic, the learning environments of students around the world drastically changed: school closures were implemented in March 2020, and the classroom as a learning and social environment was dissolved. This unprecedented event had a significant impact on parents and teachers and, as consequence, on the well-being of students (Jiao et al., 2020; Letzel et al., 2020; Spinelli et al., 2020). Socioecological models of development stress the impact of learning environments on the development of children (Bronfenbrenner, 1979). School is the most important learning environment for students in addition to the family (Eccles and Roeser, 2011). These two learning environments (microsystems) are part of the mesosystem of school-aged children and they interact with each other (Bronfenbrenner, 1979). Accordingly, the development and psychological well-being of students is shaped by interactions and experiences with teachers, peers, and parents. In light of the drastic event of emergency distance learning, it is of interest to investigate how students have experienced the new learning environment, mostly shaped by their parents and teachers.

In most European countries, emergency distance learning was established at the beginning of the COVID-19 outbreak to ensure the education of students. The primary objective of this unplanned distance learning was to provide temporary access to education that was reliable and immediately available (Hodges et al., 2020). Many students were able to continue learning remotely from home with the benefit of adapted curricula and access to smart technology and internet bandwidth (Hughes, 2020). However, the short preparation time available to teachers and schools affected the quality of instruction and students’ learning opportunities (Andrew et al., 2020; Bonal and González, 2020; Kuhfeld et al., 2020). While in a substantial number of countries schools returned to classroom instruction from May 2020, some countries maintained distance learning, albeit in a more planned and structured manner (UNICEF, 2021). In line with the regulations taken in most countries, the Swiss government implemented school closures between March and May 2020, and all schools in compulsory education provided emergency distance learning arrangements. Long-term effects are expected for both planned and unplanned distance learning. However, studies have shown that unplanned emergency distance learning required a particularly high adaptation of teachers (Hodges et al., 2020) and significantly affected the daily routines of students and their parents (Spinelli et al., 2020; Viner et al., 2020).

Although the COVID-19 pandemic has impacted everyone, children and adolescents are a particularly vulnerable group. Studies have reported parents’ observations of emotional and behavioral problems (i.e., anxiety, irritability, distraction) in their children and adolescents due to the pandemic (Jiao et al., 2020; Spinelli et al., 2020). Other studies have found significant effects of emergency distance learning in compulsory education on students’ academic achievement gains (Tomasik et al., 2020). However, less is known about students’ perspectives and, more specifically, about their perception of emergency distance learning. Gaining knowledge of their perceptions can help in understanding the impact of this unprecedented learning arrangement on their subjective well-being and in predicting the psychological effects of the implementation of distance learning in future emergency situations. In addition, there is a lack of studies on the impact of the COVID-19 pandemic considering multiple informants. Using data from multiple informants allows for a more comprehensive understanding of the situation. Further, it is important to stress that there is a lack of comprehensive theoretical models describing the novel situation of emergency distance learning during pandemics. However, certain theoretical approaches can help in understanding some of the processes involved. In this study, the aim was to evaluate emergency distance learning in compulsory schools from the perspectives of teachers, parents, and students in Switzerland. To this end, the theoretical considerations were based on theoretical models describing processes in which teachers and parents have an impact on their students and their children, respectively.

A central characteristic of distance learning is that students learn at home. In this learning environment, students are mainly supported by their parents in their learning activities. Studies report that the responsibility and involvement of parents in their children’s learning are greater in distance learning settings than in regular classroom instruction (Hasler-Waters et al., 2014; Wößmann et al., 2020). Parents organize children’s learning, instruct their children, motivate and supervise them (Hasler-Waters et al., 2014). A study conducted in Germany on emergency distance learning, due to COVID-19, has provided further evidence for a greater parental involvement in students’ learning activities compared with regular classroom instruction (Wößmann et al., 2020). This increased parental involvement has a downside, as it generated additional stress for parents and conflicts with their children (Wößmann et al., 2020). In addition to the challenges of distance learning, parents and their children were exposed to the threat of the COVID-19 pandemic. The perceived threat can cause negative emotions in individuals, which in turn can influence their social environment (Bavel et al., 2020). For example, the perceived threat of parents may affect their children’s perception of threats. In a study from China based on parents’ reports, the results indicated that children and adolescents (6 to 18years old) showed high levels of inattention, clinging, and irritability during the COVID-19 pandemic. In particular, younger children were worried that a family member could contract the coronavirus (Jiao et al., 2020). Whether children’s behavior and perception were influenced by their parents’ perception remains unanswered. The results show, however, that the COVID-pandemic had an impact on the behavior of children and adolescents. Behavioral changes such as a significant decrease in attention can be expected to affect how well students were able to learn in distance learning during the COVID-19 pandemic.

The extent to which parents had an impact on their children’s well-being during the COVID-19 pandemic was investigated in a study conducted in Italy. Parents who had difficulties dealing with stress factors during the lockdown felt more stressed. This stress was found to be related to an increase in problem behavior in children and to have a negative impact on children’s well-being (Spinelli et al., 2020). These findings are in line with theorized models of parenting stress (Belsky, 1984; Abidin, 1992). These models suggest that parenting stress is influenced by relevant stressors, such as live events (e.g., COVID-19 pandemic). Parenting stress in turn affects their children’s adjustment and development (Abidin, 1992; Morgan et al., 2002). In this process, family resources such as high socioeconomic status are crucial. Based on this research, the threat of COVID-19 can be regarded as a stressor that affected parental stress levels during distance learning. In this unprecedented learning environment, a low socioeconomic status has been found to be an aggravating factor (Bonal and González, 2020). Parental stress in turn could have affected how students experienced the new learning environment: the more stressed their parents felt, the more negatively they perceived distance learning.

The most important difference between regular classroom instruction and distance learning is the spatial distance between students and teachers (Gorsky and Caspi, 2005). Studies have shown that in distance learning, students can make gains in academic achievement comparable to regular classroom instruction (Cavanaugh, 2001; Cavanaugh et al., 2004; Rice, 2006). Similar to regular classroom instruction, students need teachers’ guidance to effectively learn in distance learning arrangements (Lehmann, 2012). However, in distance learning, teachers have reduced possibilities to supervise and scaffold their students (Stevens and Borup, 2015). In this learning environment, students are required to work and learn with a high degree of autonomy. They also need to be able to evaluate themselves and to ask appropriate questions (Offir et al., 2003). This holds especially true for the learning arrangement of emergency distance learning, in which teachers lacked experience and had to adapt their teaching practices in a very short period of time. Research on emergency distance learning, due to the COVID-19 pandemic has shown that teacher–student interactions were reduced (Wößmann et al., 2020) and students’ ability to learn autonomously and to be motivated was found to be crucial for students’ academic performance (Pelikan et al., 2021). The level of student motivation and ability to learn autonomously thus affected how students experienced emergency distance learning. In addition, not all students had the same learning opportunities and were equally able to learn with a high degree of autonomy. In particular, younger students (Tomasik et al., 2020; Blume et al., 2021), students with migration backgrounds (Manca and Delfino, 2021), and students of families with low socioeconomic status (Bonal and González, 2020) experienced more difficulties in distance learning.

Although students learned at home, teachers continued shaping their learning environment in emergency distance learning based on their expectations of gains in academic achievement. Extensive research on teacher expectations indicates that when teachers have high expectations of achievement for their students, they provide appropriate learning opportunities and support (Brophy and Good, 1970; Babad, 1993; Jussim and Harber, 2005; Wang et al., 2019). In such a learning environment, students are likely to gain academic achievement (Brophy and Good, 1970; Friedrich et al., 2015; Wang et al., 2018). This effect of teacher expectations can be observed on an individual level: students for whom teachers hold high expectations make more gains in academic achievement than students for whom teachers hold low expectations (Kuklinski and Weinstein, 2001; Hinnant et al., 2009). The effects of teacher expectations on student academic outcomes can be explained by mechanisms of self-fulfilling prophecy (Brophy and Good, 1970). Furthermore, teacher expectations have been investigated on a classroom level (Rubie-Davies, 2007; Wang et al., 2019). Studies have shown that students enrolled in classrooms with high expectations from their teachers receive a large number of instructions, explanations, scaffolding, and feedback (e.g., Rubie-Davies, 2007). These teaching practices facilitate learning processes in classroom instruction and have a positive impact on gains in student academic achievement (Rubie-Davies et al., 2006; Hattie and Timperley, 2007). In emergency distance learning, it can be assumed that the effect of teacher expectations was similar, meaning that students were more likely to make gains in academic achievement if they were taught by teachers with high expectations.

In regular classroom instruction, the individual perception of students toward their learning environment is mainly shaped by the context of their class and school (Koth et al., 2008; Modin and Östberg, 2009). How students perceive their learning environment (e.g., school) is, in turn, related to their well-being (Gietz and McIntosh, 2014; Govorova et al., 2020). For instance, the high academic achievement of students has been found to be associated with a positive student perception of school and student satisfaction with the learning environment (DeWitz and Walsh, 2002; Gietz and McIntosh, 2014). This means that students who can perform well perceive their learning environment more positively. Furthermore, classroom-aggregated perceptions have been found to affect students. More specifically, students’ collective perception that they had access to practical teacher support with schoolwork was positively related to the well-being of the classroom as a whole (Modin and Östberg, 2009). These results indicate that students enrolled in the same class can have a collective perception of their learning environment. In the context of distance learning, the question arises as to whether a collective student perception of the learning environment can be found considering that there is a spatial distance between students and social interactions among students are reduced (Flottemesch, 2000). However, there are factors in distance learning that could cause the formation of a collective perception. For instance, an exclusive use of asynchronous communication by teachers *via* e-mail (e.g., instructions, feedback) directed to the whole class could reduce individual teacher–student interactions and could result in a homogeneous perception of the learning environment among students taught by the same teacher. However, whether a collective perception of students can be found in the context of distance learning needs to be empirically tested.

In summary, social interaction among students was reduced during distance learning, mostly to interactions with parents and to a lesser extent with teachers (Wößmann et al., 2020). The reduced social interactions with teachers required students to learn more autonomously (Offir et al., 2003; Blume et al., 2021; Pelikan et al., 2021). Students who were able to work more autonomously performed better in distance learning (Pelikan et al., 2021). Considering that academic self-efficacy is related to student satisfaction with the learning environment (DeWitz and Walsh, 2002), students who performed well in distance learning because they were able to learn autonomously can be expected to have a more positive perception of distance learning (H1). More particularly, native speakers, students of families with a high socioeconomic status, and older students in secondary school were more likely to benefit from distance learning (Bonal and González, 2020; Tomasik et al., 2020; Manca and Delfino, 2021). Furthermore, as students learned from home, the parents’ role in their children’s learning was greater than in regular classroom instruction (Hasler-Waters et al., 2014; Wößmann et al., 2020). It can be assumed that parents felt stressed by the unprecedented distance learning situation at home. Based on parenting stress models (e.g., Abidin, 1992), the stress perceived by parents due to distance learning was likely to affect students’ perception of distance learning (H2), and the perceived threat of COVID-19 was a stressor for parents which affected their stress due to distance learning (H3). In the same vein, a relationship can be assumed between the threat of COVID-19 perceived by parents and students (H4). Considering the impact that the COVID-19 pandemic has had on the behavior of children and adolescents (Jiao et al., 2020; Spinelli et al., 2020), the threat of COVID-19 perceived by students can be expected to explain their learning behavior (H5). Finally, the distance learning situation at home was likely affected by the socioeconomic status of the family and the family language. At the classroom level, it can be assumed, based on an extensive body of research on expectancy effects (e.g., Wang et al., 2018), that teacher expectations of their students’ academic achievement were significant variables in the class-aggregated academic achievement of students in distance learning (H6). Finally, in line with findings on self-efficacy, the academic achievement of the class was expected to be related to the collective perception of distance learning by the class (H7).

## Materials and Methods

### Participants and Procedure

In this study, the aim was to describe emergency distance learning during March to May 2020 in four German-speaking cantons of Switzerland. All primary and lower-secondary schools in these cantons with classes in grades 4 to 8 were asked to participate in the study. After school principals gave their consent to participate in the study, teachers could voluntarily enroll in the study. Teachers asked parents to participate in the study and to give their written informed consent for the participation of students. Data were collected retrospectively after the end of school closures with standardized online questionnaires (June/July 2020). School principals, teachers, and parents received personalized links to the questionnaires. Students filled out the questionnaires in school with a personalized link the research team provided *via* the teachers.

A total of 1,321 students (50% female) in 108 classes and 62 schools completed the student questionnaire. More than half of the participants (58%, *n*=875) were enrolled in grades 4, 5, and 6 of primary school (average age: 11.67years, *SD*=0.98). The rest of the participants (42%, *n*=641) were lower secondary school students in grade 7 and 8 (average age: 14.21years, *SD*=0.71). The majority of students indicated the instruction language of German as the language spoken at home (93%), and 7% reported another language as spoken at home.

A total of 875 parents or other reference persons completed the parent questionnaire (86% mothers, 13% fathers, 1% other adults; 58% parents of primary school students, 42% parents of lower secondary school students). Parent data was available for 66% of the students. A high percentage of parents (83%) were born in Switzerland. To measure socioeconomic status, the parents indicated their occupations. The families’ average socioeconomic status [Highest International SocioEconomic Index of Occupation Status (HISEI); Ganzeboom and Treiman, 2010] was 65.74 (*SD*=15.61, *n*=857).

Furthermore, 108 class teachers (63% female; average age: 41.2years, *SD*=11.58, *min*=24, *max*=65) from primary school (58%) and lower secondary school (42%) completed the teacher questionnaire. Teachers answered student-specific questions for 1,040 students (79% of the student sample). On average, teachers had 13.70years of work experience (*SD*=11.02, *min*=1, *max*=45). Teachers had few experiences using different digital technologies (measured by 8 items) in their classes before the period of distance learning (*n*=108, 5-point Likert scale ranging from never (1) to always (5); *M*=2.01, *S*=0.46, *min*=1.25, *max*=3.13). Their attitude towards digital technologies regarding their impact on student learning was rather positive (*n*=108, 6-point Likert scale ranging from strongly disagree (1) to strongly agree (6), Cronbach’s *α*=0.87, *M*=4.50, *S*=0.69, *min*=3, *max*=6).

For the present study, data from students, parents, and teachers were matched. A subsample of 539 students included data from all three informants (female students=50%; school level: primary school=61%, lower secondary school=39%; family HISEI: *M*=66.06, *SD*=15.18; language spoken at home: German=96%, other home language=4%). The excluded subsample had data from either one or two informants’ perspectives and comprised classes with fewer than three students participating in the study. Response rate analyses, performed with *t*-tests in SPSS 27, showed no significant differences between the subsamples in the study variables, with only one exception: one item measuring students’ perception of distance learning (“How was your experience of distance learning?”) was rated lower by students, if students, parents, or both did not fill out the questionnaire in comparison to the subsample, where all data were available. The difference was significant, *t*(1194.25)=−2.15, *p*=0.032, but with a small effect size (Cohen’s *d*=−0.121). These results show no or negligible differences in the response rates between the groups. Therefore, a subsample of 539 students nested in 83 classrooms was used in the present study.

### Measures

#### Student Variables

The students’ *perception of distance learning* was measured with two items. Students rated the question “How was your experience of distance learning?” on a 5-point-Likert scale with smileys ranging from very bad (1) to very good (5) as well as the statement “I prefer distance learning over regular classroom instruction” (Huber et al., 2020) on a 6-point Likert scale ranging from strongly disagree (1) to strongly agree (6). The mean value of the two items was *M*=3.53 (*SD*=1.10, *min*=1, *max*=5.5, *n*=537).

To measure students’ *learning behavior* in the period of distance learning, students were asked to rate two items retrospectively (Item 1: “Compared to regular classroom instruction, I was more motivated.”; Item 2: “Compared to regular classroom instruction, I concentrated more”) on a 6-point-Likert scale ranging from strongly disagree (1) to strongly agree (6). The mean value of the two items was *M*=3.25 (*SD*=0.94, *min*=1, *max*=5, *n*=532).

Students rated the *perceived threat of COVID-19* in the period of distance learning retrospectively with three items (Item 1: “I was worried about contracting the coronavirus myself.”; Item 2: “I was worried that someone in my family could contract the coronavirus.”; Item 3: “I was worried about the spread of the coronavirus in Switzerland.”). Items were adapted from Wong and Tang (2005) and rated on a 6-point-Likert scale ranging from strongly disagree (1) to strongly agree (6). The mean value of the three items was *M*=3.40 (*SD*=1.22, *min*=1, *max*=6, *n*=539, Cronbach’s *α*=0.77).

#### Parent Variables

Parents rated their *perceived threat of COVID-19* in the period of distance learning retrospectively with the same three items as students on a 6-point Likert scale ranging from strongly disagree (1) to strongly agree (6). The mean value of the three items was *M*=3.60 (*SD*=1.12, *min*=1, *max*=6, *n*=534, Cronbach’s *α*=0.84).

Parents rated the *stress due to distance learning* retrospectively with three items (Item 1: “I felt an additional burden due to the change to distance learning.”; Item 2: “Giving learning support in distance learning took up much additional time.”; Item 3: “Giving learning support in distance learning led to additional conflicts with my child.”) on a 6-point Likert scale ranging from strongly disagree (1) to strongly agree (6). The mean value of the three was *M*=3.38 (*SD*=1.30, *min*=1, *max*=6, *n*=498, Cronbach’s *α*=0.81).

#### Teacher Variables

Teachers rated their *students’ gain in academic achievement* in the period of distance learning retrospectively with one item for each student (“What was the student’s achievement gain during the period of distance learning?”) on a 6-point -Likert scale ranging from very low (1) to very large (6). The mean value was *M*=3.96 (*SD*=1.11, *min*=1, *max*=6, *n*=499). The teacher-rated student academic achievement gains were aggregated on a classroom level, resulting in one value per class.

Teachers rated their *expectation of achievement by the class* retrospectively with a single item “Compared to regular classroom instruction, I expected my students’ achievement gain during distance learning to be …” on a six-point Likert scale ranging from much smaller (1) to much larger (6). The mean value was *M*=3.35 (*SD*=0.77, *min*=1, *max*=6, *n*=83).

### Analytical Strategy

In the first step, descriptive analyses and correlations were calculated using raw data with SPSS 26. The percentage of missing values per variable ranged from 0 to 8%. The data were hierarchically structured, with students nested within classes. Multilevel modeling offers an appropriate framework to examine this hierarchical data structure (Hox et al., 2017). In a second step, the multilevel structure of the data was verified. Classroom differences for the dependent variable (i.e., student perception of distance learning) were examined by calculating intraclass correlations with the R package multilevel 2.6 (Bliese, 2016). The intraclass correlation coefficient (ICC) represents the proportion of the variance explained by the grouping structure (i.e., classroom). Coefficients greater than 10–25% are reported in educational studies (Hedges and Hedberg, 2007). In a third step, multilevel structural equation modeling was performed using the R package lavaan 0.6-5 (Rosseel, 2012; Rosseel et al., 2019). In this analysis step, only clusters with more than two units were included to prevent biased estimates (Maas and Hox, 2005). Fifteen classes with fewer than three students did not fulfill this criterion and were excluded, resulting in a sample of *n*=539 in 83 classrooms. The average classroom size was of 21.71 students (*min*=10, *max*=26). The average number of participants per class was of 6.5 students (*min*=3, *max*=16). Multilevel modeling enables the investigation of the extent to which classroom differences (between-classroom variation) in teacher expectations explain the average achievement gain of the classroom and the extent to which the collective perception of distance learning is explained by the average achievement gain in the classroom. At the individual level (within-classroom variation), the extent to which students’ perception of distance learning was explained by parents’ stress due to distance learning and students’ learning behavior was examined. In addition, students’ and parents’ perceived threat of COVID-19 were included as variables related to students’ learning behavior and parents’ stress, respectively. Finally, the school level (primary vs. secondary), student gender, family HISEI, and language reported by students as spoken at home (German or other) were included as control variables at the individual level. Family HISEI and home language were included as predictors of all variables related to distance learning (i.e., parental stress due to distance learning, students’ learning behavior, and students’ perception of distance learning). Student gender and school level were included as predictors of student variables (i.e., students’ perceived threat of COVID-19, students’ learning behaviors, and students’ perception of distance learning). Full information likelihood estimation with robust standard errors (MLR) was employed to make use of all available data. The goodness of fit for the estimated multilevel structural equation model was evaluated using the following indicators: robust chi-square value (*χ*^2^), degrees of freedom (*df*), level of significance (*p*), robust comparative fit index (CFI≥0.95), robust root mean square error of approximation (RMSEA≤0.08), and robust standardized root mean square residuals (SRMR≤0.10) at the within and between levels (Schermelleh-Engel et al., 2003).

## Results

### Bivariate and Intraclass Correlations

Bivariate correlations (Table 1) showed that students’ perception of distance learning correlated significantly with the threat of COVID-19 perceived by students (*r*=−0.12, *p*=0.005) but not by parents (*r*=0.01, *p*=0.757). The threat of COVID-19 perceived by students correlated significantly with that perceived by parents (*r*=0.25, *p*<0.001). Female students reported significantly greater levels of threat from COVID-19 than male students (*r*=−0.102, *p*=0.018), and primary school students found COVID-19 more threatening than secondary school students (*r*=−0.098, *p*=0.023). Students’ perception of distance learning was positively correlated with their learning behavior (*r*=0.66, *p*<0.001) and negatively with parents’ perceived stress due to distance learning (*r*=−0.15, *p*<0.001). Parents’ perceived stress was positively correlated with students’ gender (*r*=0.103, *p*=0.017) and was negatively correlated with students’ school level (*r*=−0.24, *p*<0.001) and family HISEI (*r*=−0.11, *p*=0.014). This means that parents of male students and primary school students, and with a lower family HISEI, reported higher stress levels than parents of female students and of secondary school students, and with a higher family HISEI. At the class level (not shown in Table 1), students’ perception of distance learning was not correlated with teachers’ expected gains in student achievement (*r*=−0.12, *p*=0.006). Students’ perceptions of distance learning correlated significantly with the average gain in achievement as rated by teachers (*r*=0.18, *p*<0.001). A significant correlation was also found between teachers’ expectations of achievement for the class and students’ average achievement gain (*r*=0.37, *p*<0.001).

**Table 1:** Zero-order correlations among study items at the individual level.

S. no.		1	2	3	4	5	6	7	8	9
1.	S: COVID	1								
2.	P: COVID	0.25^***^	1							
3.	S: Distance learning	−0.12^**^	0.01	1						
4.	S: Learning behavior	0.08	0.05	0.66^***^	1					
5.	P: Stress	0.06	0.16^***^	−0.15^***^	−0.06	1				
6.	S: Gender	−0.1^*^	−0.01	0.06	−0.05	0.1^*^	1			
7.	S: School level	−0.1^*^	0.01	0.06	−0.07	−0.24^***^	−0.05	1		
8.	S: Home language	−0.02	0.09	−0.03	−0.04	−0.02	−0.01	−0.05	1	
9.	P: Family HISEI	−0.06	−0.004	−0.003	−0.06	−0.11^*^	−0.02	0.05	−0.07	1

*N=531–539; S, student reported; P, parent reported; COVID, perceived threat of COVID-19; Distance learning, perception of distance learning; Stress, perceived stress due to distance learning; Gender: 0=female, 1=male; School level: 0=primary, 1=secondary; Home language: 0=instruction language (German), 1=other language*.

* *p<0.05 (two-tailed),*

* *p<0.01 (two-tailed);*

*** *p<0.001 (two-tailed)*.

In the next step, the multilevel structure of the dependent variable (i.e., student perception of distance learning) was tested. The ICC showed that 8% of the total variance in students’ experience of distance learning and 13% of the total variance in students’ preference for distance learning over regular classroom instruction were explained at the classroom level. Furthermore, 15% of the total variance in teacher-rated gains by students in academic achievement in distance learning was explained by the classroom level. The two items of student perception of distance learning were included as a latent level 2 variable in the multilevel structural equation model. The teacher-rated gains in academic achievement were aggregated as a manifest level 2 variable.

### Multilevel SEM

**Figure 1:**
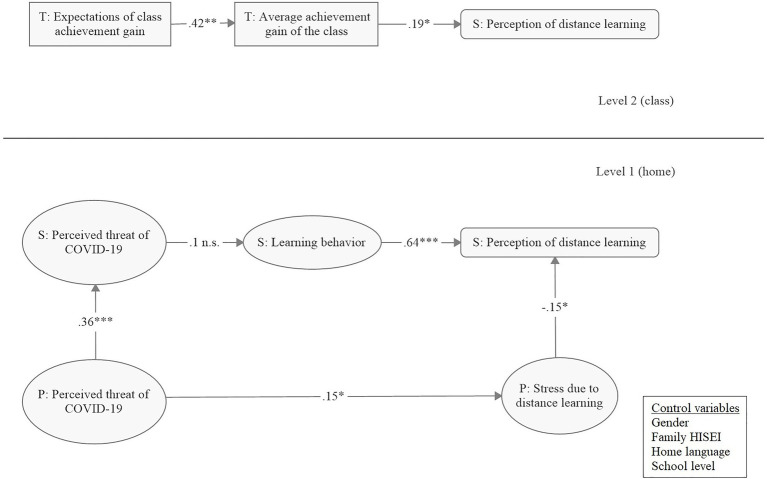
Path diagram of the hypothesized model. S=student, P=parent, T=teacher; standardized coefficients. ^*^p<0.05 (two-tailed), ^**^p<0.01 (two-tailed), ^***^p<0.001 (two-tailed).

The hypothesized multilevel model fit the data well, *χ*^2^(101, *n*=453 in 78 clusters)=296.5, *p*<0.001, CFI=0.96, RMSEA=0.07 (90% CI: 0.05, 0.08), SRMR_within_=0.04, SRMR_between_=0.05 (Figure 1). On the individual level, the results revealed that students’ perception of distance learning was significantly explained by their learning behavior (*β*=0.65, *p*=0.001) and their parents’ stress level (*β*=−0.16, *p*=0.017). Students had a more positive perception of distance learning if they were highly concentrated and motivated to learn and if their parents were less stressed in the distance learning setting. The threat of COVID-19 perceived by parents explained the parental stress due to distance learning (*β*=0.16, *p*=0.011) as well as the threat perceived by students (*β*=0.35, *p*<0.001). In other words, the more parents perceived COVID-19 as a threat, the more they felt stressed due to distance learning and the more their children felt threatened by COVID-19. However, the threat of COVID-19 that students perceived did not explain their learning behavior (*β*=0.09, *p*=0.211). Furthermore, the parents of primary school students felt more stressed than the parents of secondary school students (*β*=−0.34, *p*<0.001). Student gender explained the threat of COVID-19 perceived by students (*β*=−0.13, *p*=0.016) and their perceptions of distance learning (*β*=0.23, *p*=0.031). This means that female students felt more threatened by COVID-19 and experienced distance learning less positively than male students. All standardized factor loadings of the latent variables on level 1 ranged from 0.56 to 0.93. At the class level, teachers’ expectations about the achievement gain of their classes during distance learning explained the average achievement gain of the class (*β*=0.42, *p*=0.001): High expectations positively correlated with high gains in class-aggregated academic achievement. The collective perception of distance learning in the class was in turn explained by the average achievement gain of the class (*β*=0.15, *p*=0.037). Thus, higher gains in class-aggregated academic achievement were related to more positive collective perceptions of distance learning.

## Discussion

The goal of this study was to investigate student perceptions of emergency distance learning during the COVID-19 pandemic in light of their learning environment as shaped by their parents and teachers. Based on empirical research and theoretical models for teacher expectations and parenting stress, the impact of teachers and parents on students’ perceptions of distance learning was examined using data from all three informants and controlling for gender, family HISEI, home language, and school level. As important factors in distance learning, students’ learning behavior and average achievement gain at the classroom level were accounted for.

As hypothesized, the results showed that students experienced emergency distance learning more positively and preferred it over regular classroom instruction if they were able to learn more autonomously (i.e., more concentrated and motivated) than in regular classroom instruction, even after controlling for gender, family HISEI, home language, and school level. Thus, being able to learn successfully was positively related to students’ perception of distance learning. This study extends findings of previous studies on the effects of autonomous learning abilities. Other studies on distance learning and emergency distance learning have stressed the importance of autonomous learning for students’ academic success (Offir et al., 2003; Pelikan et al., 2021). These learning abilities are related to self-regulatory competencies, such as planning, goal setting, and self-monitoring, which have an impact on students’ academic achievement (Dent and Koenka, 2016). Thus, fostering self-regulated learning is important not only for students in regular classroom instruction but also for their academic success in learning environments that require high autonomy from learners, such as emergency distance learning. In this vein, self-regulated learning has also proven to be highly relevant for the perception of emergency distance learning (Blume et al., 2021).

Contrary to the hypothesis, students’ autonomous learning was not related to how threatened students felt by COVID-19. This result could mean that being able to learn autonomously remains unaffected by some stressors and is a resource that can help students to cope with a situation (Smith and Prior, 1995). In this case, fostering this ability is crucial. This would also mean that students lacking such stable learning abilities would be disadvantaged in distance learning arrangements and would require additional support from teachers, such as simple instructions and extensive reinforcement systems (Cavanaugh et al., 2004; Blume et al., 2021). Longitudinal studies on student learning behavior before, during, and after emergency distance learning could shed light on the extent to which stressful life events influence it. In addition, research on the effects of teacher support during emergency distance learning could provide important insights.

Parents’ impact on students’ experiences in the learning environment of emergency distance learning was confirmed by the significant relationship between parental stress due to distance learning and students’ perception of distance learning. The less parents felt stressed, the more positive their children’s experience in the novel learning arrangement was and the more they preferred distance learning over regular classroom instruction. Parental stress was in turn explained by parents’ perception of the threat of COVID-19. In addition, the threat of COVID-19 perceived by parents was related to their children’s perception of COVID-19: the higher parents estimated the threat of COVID-19, the more their children felt threatened by the pandemic. These results are in line with study findings on parental stress in the COVID-19 pandemic (Spinelli et al., 2020). They also confirm parenting stress models suggesting that life events (e.g., COVID-19 pandemic) cause parenting stress that affects the development of children (Abidin, 1992). This means that to facilitate positive experiences for students in distance learning, it is crucial to give support to their parents to cope with the stressors of live events. For instance, a way of dealing with threat is to give a sense of efficacy to deal with the situation (Bavel et al., 2020). With regard to distance learning, more interactions between teachers and students and the provision of more teacher support to students have been suggested as possible strategies to facilitate the learning of students at home (Blume et al., 2021) and to reduce parental stress in the pandemic crisis (Andrew et al., 2020; Wößmann et al., 2020). In addition, researchers in the field of distance learning have stressed the importance of clarifying the role of parents in the support of their children in distance learning settings (Hasler-Waters et al., 2014).

At the classroom level, the analyses revealed – according to the hypothesis – that teacher expectations were positively related to the average achievement gains of their classes. This finding provides evidence for the importance of teacher expectations in emergency distance learning, where teachers had to be highly adaptive to fit their teaching to the new learning environment. This is in line with research findings on teacher expectation profiles (i.e., high-expectations vs. low-expectations teachers) and on the effects of teacher expectations on student outcomes at the classroom level in classroom instruction (Wang et al., 2018, 2019). As predicted, expectations were related to students’ gains in academic achievement, and it can be assumed that teaching practices mediated this relationship between teacher expectations and student gains in academic achievement. Thus, similar to the teacher-expectation effects found in regular classroom instruction (Wang et al., 2018, 2019), students who were enrolled in a classroom with a teacher having high expectations were more likely to make gains in academic achievement during emergency distance learning than students enrolled in a class with a teacher holding lower expectations. This further indicates that the differences in terms of academic achievement gains between classes were significant and supports the observation of highly heterogeneous learning opportunities provided to students during school closures (Andrew et al., 2020).

Furthermore, the class-aggregated academic achievement gains were, as hypothesized, positively related to a collective positive perception of distance learning. In other words, students enrolled in a class that performed well during emergency distance learning were more likely to experience distance learning more positively and prefer this learning environment over regular classroom instruction. In these classrooms, teachers probably provided a learning environment that facilitated students’ ability to succeed. This mastery experience might have had an impact on collective self-efficacy beliefs at a class level (Bandura, 2000), which in turn positively affected their collective perception of emergency distance learning. This collective perception might have been enhanced through the asynchronous communication of teachers with the class, for example, *via* e-mail correspondence directed to all students jointly. However, this is an untested assumption that needs further examination. In conclusion, expecting that students are able to learn despite the difficulties caused by the pandemic and by the school closures was crucial to students’ academic success and well-being in distance learning. To form high expectations in unprecedented situations and with a lack of experience, teachers need technical and pedagogical resources from their schools, local administrations, and governments (Andrew et al., 2020).

This study provides empirical evidence for the mechanisms explaining students’ experiences during the implementation of emergency distance learning in spring 2020 using data from multiple informants. However, there are several limitations that need to be considered when interpreting the results. The first limitation concerns the cross-sectional design. All variables were collected in one wave. Unidirectional paths in the SEM were specified solely based on the theoretical rationale, and causality cannot be inferred. Longitudinal data would strengthen the significance of the results. A second limitation concerns data collection. All data were collected after the period of school closures in May 2020. Collecting data at the time of school closure was not possible as schools were busy implementing emergency distance learning. Thus, teachers, parents, and students completed their questionnaires retrospectively. As a result, information may be distorted. The third limitation is related to the variables at the classroom level. Teacher expectation was a single-item construct, and average classroom achievement was included in the structural equation model as a class-aggregated variable. Latent variables would have improved the validity of these constructs. Fourth, student gains in academic achievement were rated by teachers and not assessed with standardized tests. While teachers are very well suited to provide information on their students’ achievement gains, there is a risk that their estimations might be biased by student characteristics, such as their socioeconomic status (Wang et al., 2018). The fifth limitation concerns the COVID-19 specific variables and scales (i.e., parent stress, COVID threat, student perception of distance learning, student learning behavior). All scales were created with a limited number of items and were new, hence not validated. This was due to the short preparation time for the study, the novelty of the situation, and constraints during the period of assessment (i.e., short time period, reduced time capacities of participants). A sixth limitation is related to the control variables of socioeconomic status and home language. The percentage of families with low socioeconomic status and of families with a home language other than German was very low. This could explain why no significant relationships were found between these control variables and the study variables. The limitation section concludes with a sixth limitation on dealing with missing values. Only cases with complete information from students, parents, and teachers were analyzed; thus, the sample size was reduced to approximately one-third of the original study sample. Multilevel multiple imputations with the R package mitml 0.4-1 (Grund et al., 2016) did not result in satisfactory imputed datasets because of a large fraction of missing information (FMI). The FMI represents the loss of information due to missingness, while accounting for the amount of information retained in other variables within the data set (Madley-Dowd et al., 2019). Further studies including multiple respondents with larger samples are needed to support the use of the suggested theoretical models in emergency distance learning.

## Conclusion

To conclude, students’ perception of emergency distance learning was on one hand affected by how autonomously they could learn and by whether they were academically successful. Specifically, students with teachers holding high expectations were more likely to benefit from distance learning. This finding highlights the importance of teacher expectations, academic performance, and success even in unprecedented learning arrangements, such as emergency distance learning. On the other hand, students’ perception of the distance learning environment was related to parental stress. To ensure students’ well-being in case of the future implementation of emergency distance learning, it is crucial to reduce parental stress with social and material support from teachers, schools, and local governments. In addition, teachers need technical and pedagogical support so they can heighten their expectations of gains in academic achievement and provide students with a stimulating distance learning environment that enables them to effectively learn with greater autonomy.

## Data Availability Statement

The raw data supporting the conclusions of this article will be made available by the authors, without undue reservation.

## Ethics Statement

Ethical review and approval were not required for the study on human participants in accordance with the local legislation and institutional requirements. Written informed consent to participate in this study was provided by the participants’ legal guardian/next of kin.

## Author Contributions

AG and MN conceptualized the research. AG performed the statistical analyses, AG, EN, and JH wrote the first draft. MN contributed to the first draft, supervised the analyses, revised, and helped to finalize the manuscript. All authors organized and conducted the data collection and finalized the manuscript.

## Funding

This project was funded by the Swiss cantons of Aargau, Basel-Stadt, Basel-Landschaft, and Solothurn.

## Conflict of Interest

The authors declare that the research was conducted in the absence of any commercial or financial relationships that could be construed as a potential conflict of interest.

## Publisher’s Note

All claims expressed in this article are solely those of the authors and do not necessarily represent those of their affiliated organizations, or those of the publisher, the editors and the reviewers. Any product that may be evaluated in this article, or claim that may be made by its manufacturer, is not guaranteed or endorsed by the publisher.
